# Wavelet Analysis for Wind Fields Estimation

**DOI:** 10.3390/s100605994

**Published:** 2010-06-14

**Authors:** Gladeston C. Leite, Daniela M. Ushizima, Fátima N. S. Medeiros, Gilson G. de Lima

**Affiliations:** 1 Teleinformatics Engineering Department, Federal University of Ceará, Fortaleza, CE 60455-970, Brazil; E-Mails: matfecli@uece.br (G.C.L.); fsombra@ufc.br (F.N.S.M.); 2 Math and Visualization Groups, Lawrence Berkeley National Laboratory–LNBL, Berkeley, CA 94117, USA; 3 Division of Engineering, Brown University, Providence, RI 02912, USA; E-Mail: Gilson_Goncalves_DeLima@brown.edu

**Keywords:** SAR, wind direction, FFT, CMOD4, wind speed

## Abstract

Wind field analysis from synthetic aperture radar images allows the estimation of wind direction and speed based on image descriptors. In this paper, we propose a framework to automate wind direction retrieval based on wavelet decomposition associated with spectral processing. We extend existing undecimated wavelet transform approaches, by including à trous with B_3_ spline scaling function, in addition to other wavelet bases as Gabor and Mexican-hat. The purpose is to extract more reliable directional information, when wind speed values range from 5 to 10 ms^−1^. Using C-band empirical models, associated with the estimated directional information, we calculate local wind speed values and compare our results with QuikSCAT scatterometer data. The proposed approach has potential application in the evaluation of oil spills and wind farms.

## 1. Introduction

Oceanic images acquired by Synthetic Aperture Radar (SAR) systems enclose information of geophysical parameters of the marine environment. In particular, microwave sensitivity to surface roughness enables exploitation of SAR imagery for accurate surface wind estimation (direction and speed). SAR image analysis is a powerful tool to investigate atmospheric and marine processes at spatial scales, not attained by other space borne sensors [1]. In addition to SAR systems, radar scatterometers allow ocean surface measurements and can be especially useful in cases where the wind vector retrievals by SAR are inaccurate. Satellite-based wind mapping is a helpful tool for quick estimates of the wind conditions. This combination has proven to be more efficient than the wind climatology method, based on at least one year of accurate wind measurements. There are different approaches and applications of SAR images, we discuss some of them in this section and emphasize that the range definition of wind speed is quite controversial in the literature. Next, we will show how our method fills in the gaps of current available approaches.

Portabella *et al.* [2] proposed to retrieve wind vectors by means of combining SAR data and numerical weather prediction models as an optimal inversion method to improve SAR wind vectors estimation. In [2], the authors adopted that low winds are under 7 *ms*^−1^ when deriving wind fields from ERS-2 SAR images. Cameron *et al.* [3] combined SAR and scatterometer data to characterize wind farms and their potential energy output around coastal areas. Their investigation included the method in [2] as an alternative inversion scheme for wind vectors retrieval from SAR backscatter, using a Bayesian approach to combine trial wind vectors and weather predicted data. The method has proven to be adequate for both moderate and high winds. The range of strong (high) wind speeds according to [4], is higher than 11 *ms*^−1^.

Oil spill monitoring often uses SAR images from the ocean to extract wind vectors from streaks on the sea surface. From the wind vectors, it is possible to calculate the wind speed, which influences the visibility of slicks on the sea surface [5]. Natural films are indistinguishable from oil spills if the range of wind speeds is out of the interval 3 to 10 *ms*^−1^. However, Solberg *et al.* [6] noticed a high probability of false slicks for wind speeds less than 5 *ms*^−1^; this analysis also reported fewer dark spots from local low-wind areas when in the range between 5 and 10 *ms*^−1^. Pavlakis *et al.* reported in [7] that under low wind speed conditions, such as 3 to 7 *ms*^−1^, oil spills could yield detectable radar backscattering contrast signals. These authors assumed that medium winds are within the interval of 7 *ms*^−1^ to 13 *ms*^−1^ and high winds are above 13 *ms*^−1^.

Fichaux and Ranchin [8] calculated the orientation of wind streaks from SAR images by using a spectral domain method which consists in applying a windowed Fourier transform to the wavelet coefficients obtained from a radar image to recover the wind direction. This spectral approach used the fast Fourier transform algorithm (FFT) to search for the dominant direction of wind streaks. These directions are based on the position of the two maximal of the Fourier spectrum computed on a second-level wavelet coefficient image [9].

Instead of retrieving wind parameters using spectral methods, it is possible to run spatial domain algorithms [10,11], as the decimated wavelet transform [7], which allows feature extraction from local histograms of the image gradient direction. Among several spatial domain methods, a widely-used method is the local gradient (LG) [12], capable of retrieving wind direction using local gradients derived from smoothed amplitude images. According to [11] the LG algorithm is less efficient and tends to fail in areas characterized by a low-speed wind field where the estimates tend to be significantly non homogeneous. The main limitation of spatial algorithms is the dependence on wind rows associated with atmospheric boundary layer roll vortices in the SAR image, an approach that often requires human intervention.

Ceccarelli *et al.* [11] proposed a texture based approach for wind detection in the ocean and showed results that are more robust to noise than standard and optimized LG algorithms. This method explored the advantages of both the spectral method and the local gradient, by using a localized filtering-based approach, combining both the spatial and the frequency domains. It consisted in extracting the preferred orientation of textural patterns in the SAR image rather than from its respective energy variation.

Du *et al.* [7] introduced a method in the wavelet domain for wind direction retrieval, which could quantitatively describe the image streaks through texture information detected from the vertical wavelet coefficients within a Haar wavelet decomposition. Moreover, they have suggested that different wavelet basis functions may lead to slightly different results.

These previous algorithms consider wind speed estimation from SAR images, including scatterometer wind retrieval models such as the C-band model (CMOD) series for vertical polarization radars in transmit and receive (VV) mode, which require a well-calibrated image. The wind direction is an important input parameter for these models and it is used in [7,13–15] for wind speed estimation from SAR images. Our paper assesses these algorithms by using wind speed results from three CMOD-based models available in the literature and presents comparison among them with the QuikSCAT measures.

We extend the method introduced by Fichaux and Ranchin in [8], by improving the algorithm to detect wind direction on coastal region with wind speed within the range of 5 to 10 *ms*^−1^. Our algorithm takes a SAR image as input, decomposes it by using wavelet functions, transforms the wavelet coefficients into their spectral version and finally detects peaks in the spectrum domain to recover the orientation of the streaks. The motivation for choosing undecimated wavelets is: Mexican-hat presents suitable selectivity in position and the Gabor wavelet can be tuned to detect directional features. Our algorithm estimates the wind direction using the Fourier spectrum, although the wavelet transform provides good localization in both spatial and spectral domains. Our method takes the wavelet coefficients of the decomposed SAR image as input to peak detection using spectral energy, while it attenuates the undesirable high frequencies and maintains the main spectral energy, located perpendicular to the orientation of streaks [16]. The image decomposition by wavelets enables detection of wind streaks at a certain spatial scale and later identification of wind orientation and wind speed estimation.

This paper is organized as follows: Section 2 describes the SAR data, Section 3 presents the basic concepts of wavelet transforms to retrieve wind directions from satellite SAR data. It also describes models for wind speed estimation from SAR images with HH polarization. In Section 4, we compare the results from processing SAR images using different methods to extract wind vectors with satellite scatterometer data. Discussions about the contribution of proposed framework are in Section 5

## 2. SAR Images and QuikSCAT Data

**Figure 1.**
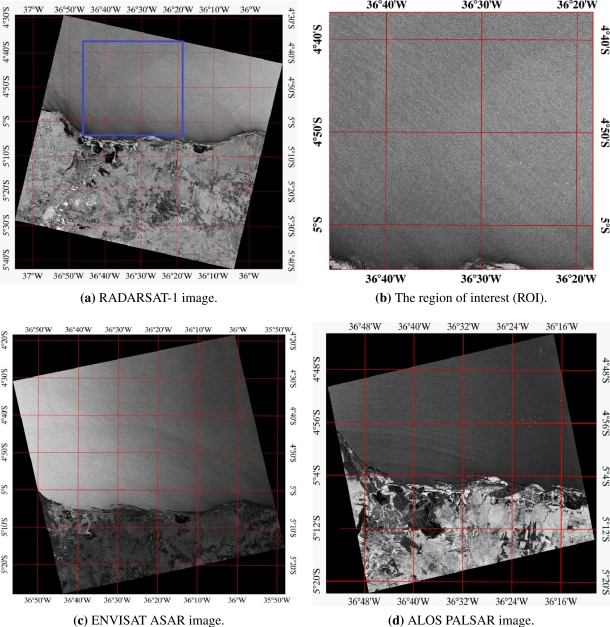
SAR images over the coast of Rio Grande do Norte, Northeast Brazil. (a) RADARSAT-1 SAR, acquired on September 29, 2006 with HH polarization. (b) Extract of the SAR image (4096 × 4096 pixels) referenced in latitude and longitude (decimal degrees) representing 51.2 × 51.2 *km*. (c) ENVISAT ASAR, acquired on February 01, 2005 with HH polarization. (d) ALOS PALSAR, acquired on July 20, 2007 with HH polarization.

We address SAR data from the RADARSAT-1, ENVISAT and ALOS PALSAR satellites, which images were acquired over the coast of Rio Grande do Norte (RN), Brazil. The Canadian satellite RADARSAT-1 acquires SAR images over the oceans on a continuous basis to support measures of geophysical parameters such as ocean surface winds. The SAR system aboard the RADARSAT-1 satellite [17] is a right looking radar, which acquires images at C-band (5.3 GHz) and at horizontal (HH) polarization in transmit and receive modes. It operates at moderate incidence angle between 10° and 59°, a swath width of up to 500 *km* and with a range of 8 to 100 *m* in resolution. RADARSAT-1 images were acquired in the standard mode, beam mode: SAR Standard 2, 100 *km* swath width. The SAR image displayed in Figure 1a was captured on September 29, 2006, at 8 : 07 a.m, with a radar incidence angle of 27.291° and with pixel size of 12.5 *m* by 12.5 *m*, corresponding to a region of the coast of Rio Grande do Norte (RN), Brazil. Figure 1b presents the region of interest (ROI) extracted from the RADARSAT-1 SAR image displayed in Figure 1a.

The advanced SAR (ASAR) aboard the European satellite ENVISAT operates in the C-band (5.34 GHz) and, in contrast to the RADARSAT-1 satellite, at both vertical (VV) and horizontal (HH) polarization in transmitting and receiving. For the following study ASAR data were acquired at HH polarization in transmitting and receiving modes. Figure 1c shows the ENVISAT ASAR image which covers the same area. This SAR image was captured on February 01, 2005 with HH polarization.

In January 24, 2006, the Japan Aerospace Exploration Agency launched the Advanced Land Observing Satellite (ALOS), which carries the Phased-Array L-Band Synthetic Aperture Radar (PALSAR). PALSAR is an active microwave sensor, which is not affected by weather conditions and operable both daytime and nighttime [18]. PALSAR is a full polarimetric (multi-polarization) system which acquires images in HH, HV, VV and VH polarization. The ALOS images (e.g., Figure 1d) were acquired in the PALSAR Fine Single, which cover areas of 40–70 *km* with pixel size of 12.5 *m* by 12.5 *m* and HH polarization.

Table 1 summarizes the SAR images information, regarding six RADARSAT-1 images, four ENVISAT images and four ALOS PALSAR images, used to validate the new wind-retrieval algorithm throughout this paper.

A different source of information came from the satellite QuikSCAT, launched on June 19, 1999. It contains the instrument SeaWinds, which measures near-surface wind speed and wind direction at 25 *km* resolution. The wind accuracy from QuikSCAT is stated to be 2.0 *ms*^−1^ in wind speed and 20° in wind direction. This accuracy depends on the distance from the shore, wind speed range and atmospheric conditions [19].

**Figure 2.**
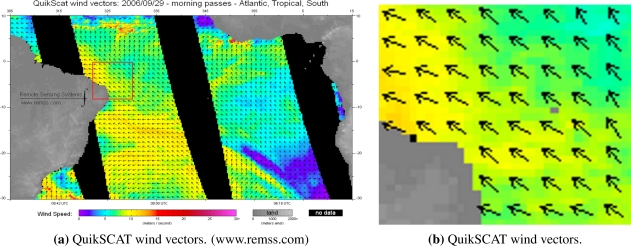
(a) QuikSCAT wind direction and wind speed estimation on September 29, 2006. (b) QuikSCAT over ROI.

The QuikSCAT daily data is a matrix of dimensions 1,440 × 720 × 4 × 2, where the first index represents longitude (from 0° to 360°), second index is latitude (from −90° to 90°), third index is UTC time, wind speed (*ms*^−1^), wind direction (degrees) and rain flag, respectively, and fourth index is ascending or descending orbit. Figure 2a shows QuikSCAT wind vectors over Atlantic, Tropical, South extracted on September 29, 2006. The first cell of the matrix is at longitude 0.125° E and in latitude −89.875°, in the 0.25° × 0.25° space resolution. The QuikSCAT wind speed is relative to a height of 10 *m* above sea level and to a neutral atmospheric stability [20]. The wind direction data follows the oceanographic convention, indicating the direction the wind blows, and are used as input variables in C-band models for calculating the wind speed.

Figure 1b and Figure 2b show our SAR and QuikSCAT data, used to retrieve the direction and speed variables over the RN coast in different dates. According to the scatterometer measurements, the wind speed values in these areas ranged from 4 to 11 *ms*^−1^ (see Table 1). From the available data set (about 14 SAR images), 5 images were selected with time difference between 7 and 12 hours, 4 with approximately 4 hours, and 3 with less than 1 hour. The interpretation of QuikSCAT region of interest relies on geographic coordinate transformation, according to Equations 2 and 3.

(1) longitude=360−longitude

(2) $$\mathrm{column}=\frac{\mathrm{longitude}+0.125}{0.25}$$

(3) $$\mathrm{row}=\frac{\mathrm{latitude}+90.125}{0.25}$$

where the longitude and latitude variables are in degrees. The acquired matrices of QuikSCAT, with wind direction and speed, are used to evaluate our direction results.

## 3. Methods

Wind field retrieval from SAR images depends upon both wind direction and speed calculation. Such information can be acquired from one or more sources as: (a) measurements of other instruments (scatterometer, buoys, *etc.*), (b) meteorological models, (c) wind-induced streaks if evident in the SAR images or from intelligent image processing remote systems. We review standard methods to be compared with the proposed approach: we summarize two methods to estimate wind direction (Subsections 3.1 and 3.2), both using wind-induced streaks from SAR images. Subsection 3.3 summarizes C-band models to estimate wind speed. Finally in Subsection 3.4, we present undecimated methods, describing the foundations of the proposed approach and our contributions to previous work.

### 3.1. The WDWaT Method for Wind Direction Estimation

Wind direction retrieval is based on the measurement of texture features from SAR images of the ocean. Each texture feature is a scalar value, computed from a whole image or a sub-scene, which characterizes the grey-level variation within the immediate area. The wind direction estimation from wavelet transform (WDWaT) is based on decimated wavelet transforms [21]. Du *et al.* in [7] introduced this approach for estimating the relative strength of the streaks in SAR images, by deriving the maximum of the standard deviations of the mean cross section (*MStdM*) as a detection criterion.

The WDWaT algorithm provides a multiscale texture analysis and identifies subscenes of weak directional features. It can quantitatively describe image streaks through the standard deviation of the mean cross section (*StdM*) of vertical details within a wavelet decomposition [7]. The cross-section mean of the area of interest is obtained by computing the mean value of each column in a vertical direction. When the image is rotated through 180° with a given rotation interval, the mean value of the cross section at different angles are obtained (rotation angle $\frac{180}{n}$). The choice of *n* depends on the accuracy of the required estimation.

The *MStdM* and the average of the standard deviations of the mean cross section (*AvStdM*) of these curves are calculated as follows:

$$\begin{array}{l} \mathrm{M StdM}=\max[\mathrm{StdM}(1),\;\mathrm{StdM}(2),\;\cdots \;,\;\mathrm{StdM}(n)]\\ \mathrm{AvStdM}=\mathrm{average}\;[\mathrm{StdM}(1),\;\mathrm{StdM}(2)\;,\cdots ,\;\mathrm{StdM}(n)] \end{array}$$

where *StdM*(*i*) is the standard deviation of the mean cross section for the *i-th* rotation angle. The factor *K* to describe the strength of the directional features [7] is given by:

(4) $$K=\frac{\mathrm{M StdM}}{\mathrm{AvStdM}}\geq 1.$$

The factor *K* is fundamental to determine the optimal spatial scale for the directional estimation of texture features. Also, *K* can be used to make quality-control decisions [7]. The higher the value of *K*, the stronger the directional features in the image.

### 3.2. The LG-method for Wind Direction Estimation

Koch proposed in [12] the local gradient method (LG) which divides an image into sub-images, depending on the space grid over which the wind characterization is requested; then image operators are applied to the images to produce a map of valid points, on which the local gradient directions are computed. The LG method consists of three steps to derive wind direction. In the first step, the SAR image is smoothed and reduced to pixel size of 100, 200, and 400 *m*. From each of these images, local directions, defined by the normal to the local gradient, are computed leaving a 180° ambiguity. In the second step, all the pixels that are effected by non-wind-induced features are masked and excluded from further analysis. Finally, only the most frequent directions in a predefined grid cell are selected from all of the resulting directions [22]. The wind direction, assumed to be parallel to the wind streaks, is thus perpendicular to the direction of the gradient. The direction of the gradient is the direction of highest increase of a streak [13]. The LG method has in principle the advantage of being more localized, allowing the wind direction estimation at higher resolution. However, the presence of noise requires large windows for the local histogram in order to obtain reliable estimate [11]. The algorithm may extract features unrelated to the wind and ignore these points while working in the spatial domain by evaluating the local gradients.

### 3.3. Wind Speed Retrieval Models for Assessment and Comparison Purposes

We estimate the wind speed from RADARSAT-1 data using three C-band models: CMOD4 [23], CMOD-IFR2 [24] and CMOD5 [25,26]. The QuikSCAT scatterometer is well collocated in time with RADARSAT-1 orbit, and ERS-2 is close to the ENVISAT orbit [19]. This paper deals with CMOD models, therefore wind speeds are estimated from RADARSAT-1 images. As the ALOS satellite operates in L-Band, wind speed estimation is not performed for the ALOS PALSAR images.

The algorithm based on the CMOD4 model was originally developed with three types of Earth observation data: the scatterometer data (ERS-1), the wind vectors from the European Centre for Medium Range Weather Forecasts (ECMWF) for surface wind analysis, and the wind and wave information from the National Oceanic and Atmospheric Administration (NOAA) wind and wave buoys, respectively [27]. The CMOD-IFR2 is very similar to the CMOD4 model, and most algorithms for C-band SAR wind retrieval are based on them [16].

The precise wind direction information is necessary to estimate accurate wind speed when using CMOD models and Equation (5). Under certain circumstances, it is possible to extract wind direction directly from SAR images. In this paper we provide a set of experiments to assess the effect of wind direction in the wind speed estimation by using CMOD models.

Wind speed retrieval relies on an empirical model function, which relates the normalized radar cross section (NRCS) of the ocean surface *σ_o_* to the local near-surface wind speed *v*, wind direction versus antenna look direction Φ, and incidence angle *θ*. The general form of the function is given by

(5) $$\sigma_{o}=B0{\left(1+B1\cos(\Phi )+B2\cos(2\Phi )\right)}^{p}$$

where *B*0, *B*1 and *B*2 are coefficients that depend on the incidence angle, wind speed, radar frequency and polarization and *p* ∈ ℝ. For the C-band, these coefficients were determined empirically by evaluating ERS-1 data, which operates at the C-band with VV polarization, and wind fields from the ECMWF [13]. These functions are applicable for wind-speed retrieval from VV-polarized SAR images. The CMOD4 and CMOD-IFR2 have been applied successfully to ERS-1 and ERS-2 images [16].

Particularly RADARSAT-1, the SAR system operates at C-band but with HH polarization, then the CMOD models cannot be directly used as they are acquired. This happens due to *σ_o_* decrease as the incidence angle increases and the increasing wind speed sensitivity to the error from the wind direction.

Thompson, Elfouhaily, and Chapron [28] derived an empirical expression for the polarization ratio to obtain an approximate form for the HH polarization backscatter from RADARSAT-1. This hybrid expression is given by:

(6) $$\mathrm{PR}=\frac{\sigma_{o}^{\mathrm{HH}}}{\sigma_{o}^{\mathrm{VV}}}$$

where $\sigma_{o}^{\mathrm{HH}}$ and $\sigma_{o}^{\mathrm{VV}}$ are the HH and VV-polarized NRCS, respectively. Different *PR* functions have been suggested in the literature [13,24,28]. We obtained the wind directions from SAR images with the spectral algorithm and the values are used as inputs to the CMOD models. Next, we compare these results with those computed from QuikSCAT.

### 3.4. Undecimated Wavelets

Undecimated wavelet transforms (UWT) or stationary wavelet transform is a shift invariant transformation, relevant to detect wind direction in SAR images. We use the UWT to decompose a SAR image into wavelet coefficients to emphasize details in different scales of the image. The wavelet coefficients are the input to the spectral method, followed by the identification of the maximum values in the Fourier spectrum. The next sections present different versions of the UWT algorithm, using different basis functions.

In UWT decomposition, the number of the wavelet coefficients does not decrease among the scales. This additional information can be very useful for better analysis and understanding of the signal. The translation-invariant property of the undecimated wavelet transforms is relevant to the feature-extraction [29], particularly in the detection of streaks.

#### (A) The à Trous Wavelet Transform

The à trous (with holes) algorithm decomposes a signal without subsampling, *i.e.,* no decimation step is undertaken and in each projection only the filters are dilated [29–33]. This transform was successfully used by Fichaux and Ranchin [8] over a triangular function. Our paper includes the Mexican-hat and Gabor undecimated wavelet transforms and the *B*_3_-spline basis.

The à trous algorithm allows the separation of low-frequency information (approximation) from high-frequency information (wavelet coefficients or detail coefficients). This UWT can be interpreted as a frequency decomposition with each set presenting a different spatial orientation. According to Bijaoui *et al.* [33], two scaling functions lead to a piecewise linear interpolation: the triangular function and the *B*_3_-spline.

The main reason to choose the à trous algorithm for this application is the information redundancy between decomposition scales observed in the inherent gradual blurring effect. This algorithm consists in convolving the original signal, *s*(*k*), with a filter *h* which is interpolated by 2^*j*−1^ zeros at each decomposition scale *j*. The reconstruction of the original signal *s*(*k*) is obtained by adding the last smoothed signal *s_N_*(*k*) with the set of wavelet coefficients [34],

(7) $$s(k)=s_{N}\;(k)+\sum_{j=1}^{N}w_{j}\;(k)$$

where *N* is the number of all wavelet scales.

#### (B) The Gabor Wavelet Transform

The Gabor wavelet is a complex-valued wavelet which obtains the optimal localization in spatial and frequency domains, simultaneously. Furthermore, the Gabor wavelet is directional and capable of tuning to specific frequencies, thus allowing it to be adjusted for streak enhancement and orientation detection. The 2-D Gabor function *g*(*x*, *y*) is defined as [35]:

(8) $$g(x,\;y)=\left(\frac{1}{2\pi \sigma_{x}\;\sigma_{y}}\right)e^{-\left[\pi \left(\frac{{\left(x-x_{0}\right)}^{2}}{\sigma_{x}^{2}}+\frac{{\left(y-y_{0}\right)}^{2}}{\sigma_{y}^{2}}\right)\right]}\;e^{\left[i(\xi_{0}x+\nu_{0}y)\right]}$$

where (*x*_0_, *y*_0_) is the center of the spatial domain and (*ξ*_0_, *ν*_0_) is the optimal spatial frequency of the filter in the frequency domain. Here, *σ_x_* and *σ_y_* are the standard deviations of the modulated Gaussian along *x* and *y* axes.

#### (C) The Mexican-hat Wavelet Transform

The 2-D Mexican-hat wavelet function is widely used for zero-crossing multiresolution edge detection [36] and defined as it follows [37]:

(9) $$\psi (a\vec{x})=\left(2-{\left|a\vec{x}\right|}^{2}\right)\;\text{exp}\;\;\left(-\frac{a{\vec{x}}^{2}}{2}\right)$$

where *x⃗* gives the two-dimensional coordinate of a pixel and *a* is a scale parameter which also works as the sample period of the Mexican-hat function. In spatial-frequency domain, it is written as:

(10) $${\hat{\psi }}_{H}(\vec{k})=(\vec{k}.\vec{k})e^{\left(-\frac{1}{2\vec{k}\cdot \vec{k}}\right)}$$

where *k⃗* represents the 2-D spatial-frequency variable and · is the inner product.

The 2-D Mexican-hat transform tends to be an effective band-pass filter, often used to separate different scales in the image to show their relative phase/location information. These characteristics make the 2-D Mexican-hat wavelet transform a strong candidate method in the detection of wind streaks from SAR images.

### 3.5. Proposed Spectral Algorithm for Wind Direction Estimation

**Figure 3.**
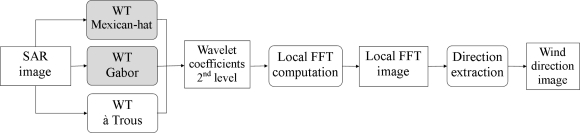
Algorithms under investigation for wind direction detection: proposed algorithms (top and center) and the Fichaux and Ranchin’s algorithm [8] (bottom).

Our method encompasses the undecimated wavelet transforms with à trous (*B*_3_-spline), Gabor and Mexican-hat, as illustrated in Figure 3. We extend the algorithm in [8] by using other wavelet transforms, which have the potential to improve the streak detection results.

The spectral method extracts the wind direction from SAR images, by applying a windowed FFT to the wavelet coefficient image to model the wind waves. The spectral algorithm considers successive sub-images of the second level coefficient image. The first level of wavelet coefficients is inadequate in our analysis because it focuses on the spatial scale ranging from 100 to 200m [8], besides being more subjected to noise. In a precision image with 100 *m* pixel size (200 *m* resolution), the image of the wavelet coefficients represents spatial scales in 200–400 *m*. In many cases the wind-induced waves are clearly visible in SAR images as almost linear patterns, called wind streaks, representing scales between 200 and 1,600 *m*, where wind-induced phenomena aligned with the wind direction are most likely to occur [38]. The position of the maximum Fourier spectrum calculated from the wavelet coefficients (second level) indicates the wind directions.

We apply a local FFT to a SAR image to extract the wind direction with a grid size of 250 × 250 pixels, equals to a 25 × 25 *km* grid cell. This grid cell corresponds to the QuikSCAT resolution. For assessment purpose, we compare the wind direction information estimated by the FFT algorithm with two algorithms available in the literature and described in the next section and also with QuikSCAT data.

We estimate direction with Gabor wavelets by rotating the Gabor function (Equation (8) at steps of 10°. There are a total of *M* different frequencies and *N* different orientations, resulting in *M* × *N* coefficients for each image pixel (*x*, *y*). Equations (11) and (12) refer to the rotation property, as follows:

(11) $$g_{\mathrm{mn}}\;(x,\;y)=g(x^{\prime },\;y^{\prime })$$

where *m* ∈ [1, *M*], *n* ∈ [1, *N*] and *g*(*x, y*) refers to Equation 8. The rotation matrix is given by

(12) $$\left[\begin{array}{c} x^{\prime }\\ y^{\prime } \end{array}\right]=\left[\begin{array}{cc} \text{cos}\;\theta_{n} & \text{sin}\;\theta_{n}\\ -\text{sin}\;\theta_{n} & \text{cos}\;\theta_{n} \end{array}\right]\;\left[\begin{array}{c} x\\ y \end{array}\right],\;\;\;\theta_{n}=\frac{n\pi }{N}.$$

By convolving an image *I*(*x*, *y*) with Gabor wavelets, the Gabor transformed image can be defined as:

(13) $$\hat{I}(x,\;y,\;m,\;n)=\int I\;(x^{\prime },\;y^{\prime })g_{\mathrm{mn}}\;(x-x^{\prime },\;y-y^{\prime }){\mathrm{dx}}^{\prime }{\mathrm{dy}}^{\prime }.$$

In this paper we are interested in the response with the maximum magnitude over all possible orientations, namely:

(14) $$T=\underset{\theta }{\text{max}}\;|\;\hat{I}\;(x,\;y,\;m,\;n)|$$

**Figure 4.**
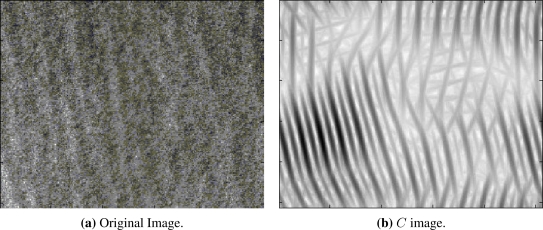
(a) Original SAR image *I*. (b) Image of the dominant directions of the induced streaks in the ocean detected by the Gabor wavelet.

After calculating the *T* image with the maximum magnitude response in all directions, we obtain the coefficient image *C* by subtracting *T* from *I*, as illustrated in Figure 4b. Finally, the wind direction estimation is calculated by applying the FFT to the image *C*, resulting in a new representation of the imagette, with streak contrast enhancement.

Our approach of the Mexican-hat wavelets for wind direction retrieval consists in convolving the function in Equation (9) with the SAR image, followed by the difference between the SAR image and the convolution results. Then, the spectral algorithm computes the wind direction from the imagettes of the coefficient images.

## 4. Results

This section presents the outcomes of 7 different techniques for wind direction calculation. We compare 3 standard methods with our 3 proposed approaches, by using QuikSCAT direction values as the gold standard. Next, we calculate wind speeds, using only the wind directions obtained from FFT-based methods, yet checking the agreement with QuikSCAT speed values.

**Figure 5.**
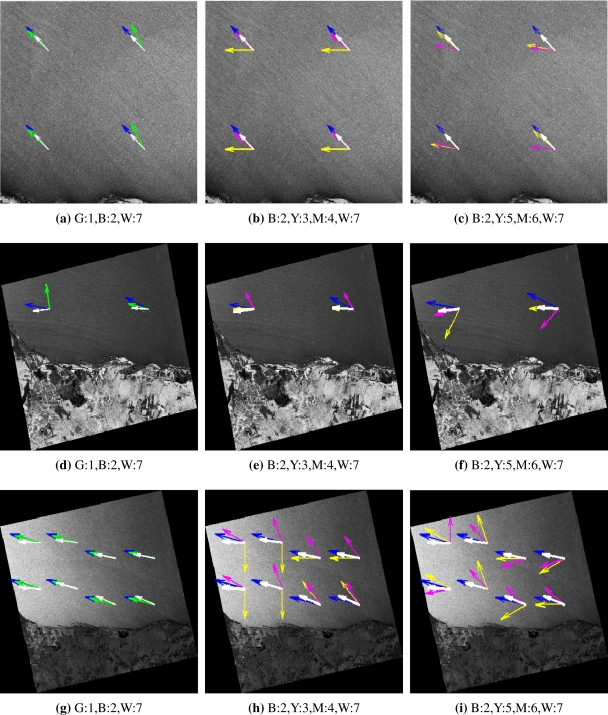
Wind direction vectors from 3 different SAR images: (a–c) RADARSAT-1 SAR image, on September 29, 2006, (d–f) ALOS PALSAR image, on July 20, 2007 and (g–i) ENVISAT ASAR image, acquired on February 01, 2005. White arrows indicate the ground-truth value, from QuikSCAT in all images; color-method associations appear on the label of each image.

We test the algorithms with a set of fourteen SAR images, which refer to the same area, laying out between 4°30′*S* and 5°40′*S* in latitude and 35°50′*W* and 37°00′*W* in longitude, in different dates and weather conditions. Each image is split into imagettes before the calculation of respective wind direction vectors. In Figure 5(a), one imagette corresponds to approximately one quadrant of the image, from which a direction vector is calculated for each method. Imagettes are 250 × 250 pixel subimages from the SAR images and we use a total of 41 imagettes.

For an easier reading of Figure 5, we label the methods numerically such as: (1) UWT with triangular base, (2) UWT with *B*_3_-spline, (3) UWT with Gabor, (4) UWT with Mexican-hat, (5) WDWaT, (6) LG and (7) QuikSCAT. This figure illustrates the wind direction using each of the 7 methods for 3 SAR images; each of these images contains a different number of valid imagettes. The color code for the direction vectors can be blue (B), green (G), yellow (Y), magenta (M) and white (W), and they correspond to one method, in conjunction to a numerical identifier as pointed out above. As an example, the code G:1, indicates green arrows, which represent the wind directions calculated by method (1). Notice that each row of Figure 5 shows the same SAR image, but with arrows representing the wind direction result of different methods over each imagette. Table 2 presents the mean and standard deviation of wind direction for the different methods, with bold numbers indicating high similarity to the QuikSCAT values.

In order to evaluate the results of the spectral algorithm over the detail images obtained from the wavelet decompositions, we adopt the following empirical parameters: the Gabor wavelet uses: *σ_x_* = *σ_y_* = 6.95, *ξ*_0_ = 3.14 and *ν*_0_ = 0, tuned according to the dimension of the streaks (200 to 1,600m) in our dataset. The Mexican-hat wavelet uses parameter *a* set to 0.3*π*, which resulted in noise suppression and streak recovery.

Before comparing the wind fields between the scatterometer and the SAR-derived results, we filter the input data following the criteria: (a) removal of rain-contaminated areas due to scatterometer data to be less accurate in such circumstances and (b) total overlay of the scatterometer resolution cell (25 *km*) within the given SAR scene [19]. Although SAR images are independent of weather, rainy areas in scatterometer data can result in erroneous cross track vectors and/or incorrect high speed values [20]. Furthermore, we separate the imagettes in two groups, according to the speed values from QuikSCAT: (a) data set consisting of all 41 imagettes (b) data set consisting of only imagettes with wind speeds *<* 10 *ms*^−1^ (32 imagettes).

**Figure 6.**
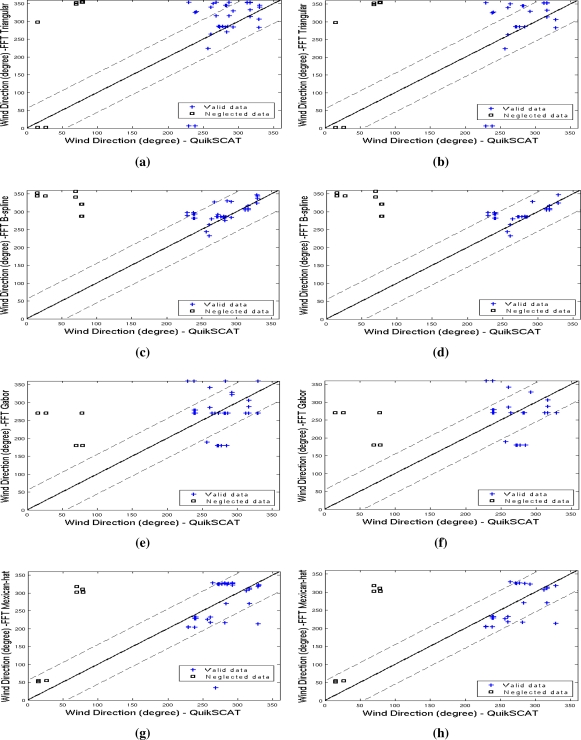
Comparison between QuikSCAT (abscissa) and SAR-based methods (ordinate) for two data sets: (a, c, e, g) after removing the low-confidence (rain cells) from QuikSCAT data and (b, d, f, h) regions with wind speeds less than 10*ms*^−1^; the FFT methods differ from their wavelet decompositions: à trous, triangular base (a, b), à trous *B*_3_-spline (c, d), Mexican-hat (e, f) and Gabor (g, h).

After calculating the wind direction over each imagette, we illustrate the direction from each imagette against its correspondent QuikSCAT value in Figure 6. In this figure, imagettes wind vectors appear in blue crosses and QuikSCAT-rain flagged regions in black boxes as neglected data. The main range of wind direction variation is highlighted by dashed lines. Our results (see Table 2) are consistent with wind directions in the northern coastline of Rio Grande do Norte, Northeastern Brazil (around 36°W and 5°S), where the wind blows from East during August to April, and from Northeast during May to July [39]. Indeed, the predominant wind direction in this area is from East and according to the geographic convention it is expected to be between 202.5° and 292.5°. In addition, Oliveira *et al.* [39] also reported that from March to June, the mean wind speed is expected to be 4.8 *ms*^−1^ while between August and December, the winds are expected to be stronger (around 9.0 *ms*^−1^).

We use statistical descriptors as the bias, root mean square error (RMSE), correlation, standard deviation, mean and maximum values in Table 3, to interpret the goodness-of-fit among the spectral methods, showed in Figure 6. Bold numbers indicate high correlation and low RMSE occurrence, simultaneously, an indicator of agreement between the spectral method and the QuikSCAT output. Based on such analysis, we notice that the spectral methods presented best performance, particularly for imagettes with wind speeds up to 10 *ms*^−1^.

Certainly, the 2-D Mexican-hat wavelet characteristics as continuity and axis symmetry have played an important role in extracting structures as streaks. This method detects the highest and lowest backscatter structures in the SAR images, providing the best results in our experiments. Also, we observe that the à trous wavelet transform decomposition with *B*_3_-spline base function achieves comparable results to the 2-D Mexican-hat results. Table 3 shows that it outperforms the other methods regarding the RMSE and correlation measures, particularly for data set with wind speeds up to 10 *ms*^−1^. Wind directions estimated by this method are highly correlated (0.61) with QuikSCAT data and thus present the lowest RMSE (31.15°) and standard deviation (23.31°).

The Gabor wavelet transform combined with the spectral method performs poorly in comparison with other methods if we look at the bias, RMSE and correlation, as shown in Table 3. Notice that the estimated directions are low correlated (−0.11 and −0.22) with QuikSCAT and the RMSE values (60.68° and 69°) indicate that the Gabor function misses most of the streak patterns from SAR images.

In accordance to the results reported by Fichaux and Ranchin [8], the à trous wavelet transform decomposition with a triangular base function, performs well in areas of high wind speeds (above 9–10 *ms*^−1^) and it is less accurate when performed in areas of low to moderate wind speeds (4–9 *ms*^−1^).

Henceforth, our investigation focuses on the two best spectral methods: à trous with *B*_3_-spline and Mexican-hat. We use wind direction results of these two methods as the inputs to CMOD models for wind speed estimation. Thus, for each imagette we compare the CMOD results with the corresponding QuikSCAT speed data.

**Figure 7.**
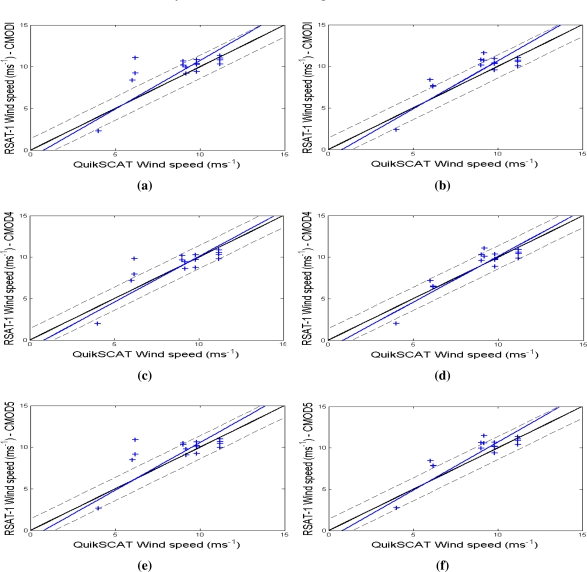
Comparison of wind speed retrieval results and QuikSCAT scatterometer winds. (a, c, e) Wind direction estimated by the FFT method using *B*_3_-spline function. (b, d, f) Wind direction estimated by the FFT method using Mexican-hat function.

Figure 7 and Table 4 provide data to compare RADARSAT-1 and QuikSCAT wind speeds. Under low wind speeds, secondary factors can affect the backscatter from the ocean such as meteorological phenomena and oceanic phenomena, causing backscattering variations for the same wind intensity [19]. The largest differences between the 3 C-band models occur at wind speeds above 10 *ms*^−1^. At moderate wind speeds they agree fairly well. The CMOD-IFR2 and CMOD5 models are very similar to each other. The main difference occurs at very high wind speeds *>*20 *ms*^−1^, where CMOD5 tends to output higher winds [13].

We perform the experiments by using the best wind direction results as inputs to C-band models to reduce estimate errors. In areas of low to moderate wind speeds the approximation of estimated speeds and QuikSCAT data was better for CMOD4 with the lowest RMSE values (1.34 *ms*^−1^ and 0.99 *ms*^−1^). Table 4 displays the estimated speeds with CMOD4 for the RADARSAT-1 SAR standard images. They are highly correlated (0.79 and 0.9) with QuikSCAT data.

The comparison with respect to the different CMOD models is performed using the wind directions resulting from the FFT algorithm with à trous wavelet (*B*_3_-spline) and FFT algorithm with Mexican-hat wavelet. In this paper, we apply the *PR* model called Elfouhaily scattering to estimate the NRCS for SAR images with the HH polarization, as suggested in [27]. Such a model allows estimation of wind speed in fairly agreement with wind speed values at several meteorological observation stations. Table 4 displays that CMOD4 outperformed the other C-band based models concerning RMSE and correlation values. It implies that the estimated speed values are close to the QuikSCAT values. At low to moderate wind speed values, CMOD4 is the best choice to retrieve SAR wind speed in high resolution SAR images acquired at C-band [26]. However, especially at high wind speed, CMOD4 underestimates the wind speeds significantly. Also CMOD-IFR2 and CMOD5 output better estimations at high wind speed values, but still underestimate the wind speed [13].

## 5. Conclusions

We proposed a framework to retrieve wind direction from RADARSAT-1 and ENVISAT ASAR images acquired with HH polarization in transmitting and receiving at C-band and from ALOS PALSAR images collected at L-band and with HH polarization. Wind speeds were retrieved from RADARSAT-1 images using an empirical model that gives the dependency of the NRCS on wind speed, wind direction and incidence angle. The model was developed for the ERS-1 SCAT operating at C-band with VV polarization, and was extended to HH polarization by considering an incidence-angle-dependent polarization ratio.

Our algorithm decomposed images by applying undecimated wavelets and Fourier transforms to estimate direction of the prevailing winds in SAR images. The novel steps encompassed the Gabor and Mexican-hat undecimated wavelet transforms to derive detail images. The performance of the algorithms was compared with the LG and WDWaT methods. Furthermore, we also implemented a standard and widely-used spectral method in the literature, with a different scaling function, the *B*_3_-spline, obtaining better results, given the wind speed range under inspection.

The main difference between the Mexican-hat wavelet and the à trous algorithm with *B*_3_-spline relies on the fact that the former enhances the streak patterns, as well as the latter, but it also enhances undesirable noise and small-scale fluctuations when deriving wind fields from SAR images. It is a particular characteristic of the Mexican-hat wavelet. Both methods performed similarly when discarding imagettes containing wind speed values *>*10 *ms*^−1^. In this case, the algorithms achieved the lowest RMSE and the highest correlation values. Our investigations suggested that it was accomplished by the multiscale blurring effect, provided by the *B*_3_-spline and Mexican-hat wavelet bases, which reduced undesirable noise, and small-scale surface roughness, in the range of low to moderate wind speeds. In addition, this blurring effect preserved relevant information (e.g. streaks) for direction estimation for several scales. Our results also suggested that the wavelet coefficients, obtained with the *B*_3_-spline base function, were more suitable to characterize wind-induced streaks oriented in the wind direction in scales higher than 200 *m*. It means that the à trous decomposition with triangular function in low to moderate wind speed areas is more sensitive to small-scale roughness than *B*_3_-spline base function, as we expected.

We noticed that speckle noise caused small-scale fluctuations in the backscatter of the SAR images. This motivated our tuning of the *B*_3_-spline and Mexican-hat functions to extract wind-induced streaks and ignore surface small-scale intensity variations. It is noteworthy that the proposed method also smoothed speckle when applied to our dataset of multi-look SAR images. The combination of smoothing effect and multi-look processing, with streak pattern enhancement for wind fields estimation, improved the algorithm accuracy. Due to the ability of these masks to smooth variations of intensity at small-scales, the performance of the algorithm was superior in areas of low to moderate wind speeds in comparison with areas of high wind speeds. On the other hand, we observed that the energy of the Gabor wavelet function could have been tuned differently, probably improving wind direction estimates if considering a more extensive exploration of the parameters for better alignment with the streak patterns.

Further developments will include a larger data set to evaluate the performance of the proposed method for wind field estimation for different terrains. Preliminary tests show that SAR images of hurricanes in the Pacific Ocean could be detected using the proposed algorithm. We might extend the algorithms for application to images from storms, hurricanes, typhoons and oil spill detection.

## Figures and Tables

**Table 1.** The set of SAR images using a 12.5 m pixel size.

Satellite	Mode Beam	Orbit	Image Time UTC	Wind Conditions ^1^
RADARSAT-1	Standard 7	39713	2003/06/14 07:56	M/9.1
RADARSAT-1	Standard 2	39756	2003/06/17 08:09	M/6.3
RADARSAT-1	Standard 7	56863	2006/09/26 07:55	H/11.2
RADARSAT-1	Standard 2	56906	2006/09/29 08:07	M/9.8
RADARSAT-1	Standard 3	56906	2001/02/03 20:42	M/6.1
RADARSAT-1	Standard 6	56906	2001/02/07 07:53	L/4.0
ENVISAT	IMG	11779	2004/06/01 00:39	M/9.4
ENVISAT	IMG	15286	2005/02/01 00:38	M/9.8
ENVISAT	IMP	19566	2005/11/29 00:41	H/11.0
ENVISAT	IMP	25342	2007/01/04 12:13	M/6.9
ALOS	FBS8	7905	2007/07/20 01:16	M/10
ALOS	FBS8	12602	2008/06/06 01:13	M/8.2
ALOS	FBS8	18641	2009/07/25 01:18	H/10.5
ALOS	FBS8	19064	2009/08/23 01:16	M/9.7

1 L, low wind (< 5 *ms*^−1^); M, moderate wind (5 *ms*^−1^ < *v* < 10 *ms*^−1^); H, high wind (> 10 *ms*^−1^). Mean value of speed wind provided by QuikSCAT.

**Table 2.** Wind direction results to be compared with QuikSCAT measures.

		FFT				WDWaT	LG	

SAR images	Measures	à trous		Gabor	Hat	Haar	Gradient	QuikSCAT
		Triangular	*B*_3_-spline					

2003/06/14	Mean (°)	**352.6**	**306.2**	270	287.66	355.0	**294**	314.2
	Std. dev. (°)	**0.3**	**2.1**	0	20.4	5.8	**4.8**	2.6

2006/09/26	Mean (°)	334.3	**328.5**	**270.0**	180.84	335.0	**264.3**	277.5
	Std. dev. (°)	22.2	**1.9**	**0**	169.1	5.8	**10.4**	10.4

2006/09/29	Mean (°)	322.6	**316.3**	279.0	**311.61**	**295.0**	279.4	316.5
	Std. dev. (°)	11.1	**0.07**	10.6	**2.3**	**5.7**	4.7	0.0

2001/02/03	Mean (°)	275.76	**246.33**	272.33	225.12	303.33	**270.46**	259.5
	Std. dev. (°)	58.26	**15.84**	76.96	7.42	56.86	**0.09**	2.59

2001/02/07	Mean (°)	**328.57**	**327.99**	**327.53**	**321.67**	190	**270.35**	292.5
	Std. dev. (°)	**0**	**0**	**0**	0	0	**0**	0

2005/11/29	Mean (°)	**270**	**275.53**	360	**270**	360	241.89	283.5
	Std. dev. (°)	**0**	**0**	**0**	**0**	0	0	0

2007/01/04	Mean (°)	252.66	290.39	317.03	**217.28**	280	**254.84**	235.5
	Std. dev. (°)	152.9	6.13	46.03	**13.94**	58.06	**11.33**	5.01

2005/02/01	Mean (°)	**284.51**	**284.83**	237.83	325.74	287.5	309.49	282
	Std. dev. (°)	**1.18**	**1.22**	64.8	1.77	39.55	40.95	7.94

2007/07/20	Mean (°)	316.58	**286.04**	**270**	326.54	240	241.81	268.5
	Std. dev. (°)	47.25	**9.30**	**0**	**1.30**	42.43	21.73	6.36

2008/06/06	Mean (°)	294.44	**335.56**	270	264.92	230	209.79	328.5
	Std. dev. (°)	16.57	**16.57**	0	73.59	0	8.59	0

2009/07/25	Mean (°)	**342.17**	**340.34**	315	**321.89**	320	224.99	330
	Std. dev. (°)	**4.37**	**4.19**	63.64	**1.4**	28.28	15.34	0

2009/08/23	Mean (°)	**344.64**	**285.99**	270	243.2	340	**263.75**	282.75
	Std. dev. (°)	**0.4**	**1.31**	0	37.89	28.28	**6.05**	1.06

**Table 3.** Statistical parameters of the comparison of the scatter plot shown in Figure 6.

Measures	**Total data set (41 imagettes)**				**Only imagettes with wind speed values** < **10***ms*^−1^			
	Triangular	*B*_3_-spline	Gabor	Hat	Triangular	*B*_3_-spline	Gabor	Hat
bias (°)	19.75	**17.01**	−0.13	−12.24	19.90	**16.39**	−1.82	−**10.25**
RMSE (°)	72.13	**31.15**	60.68	63.66	82.60	**31.24**	69.00	**38.82**
correlation	0.35	**0.57**	−0.11	0.47	0.35	**0.61**	−0.22	**0.62**

std. dev. (°)	73.92	24.57	49.24	70.61	85.50	23.31	53.22	47.44
mean (°)	301.39	298.65	281.50	269.39	298.23	294.71	276.51	268.07
maximum (°)	353.54	347.28	360	328.28	353.39	347.28	360	327.46

QuikSCAT parameters								

mean (°)	281.63				278.33			
std. dev. (°)	30.54				33.50			
maximum (°)	330				328.5			

**Table 4.** Statistical parameters of the comparison of the scatter plot shown in Figure 7.

Measures	*B*_3_-spline			Mexican-hat		
	CMOD-IFR2	CMOD4	CMOD5	CMOD-IFR2	CMOD4	CMOD5
bias (*ms*^−1^)	0.79	**0.12**	0.64	0.63	**0.06**	0.68
RMSE (*ms*^−1^)	1.75	**1.34**	1.71	1.34	**0.99**	1.26
correlation	0.72	**0.79**	0.69	0.85	**0.90**	0.87

std. dev. (*ms*^−1^)	2.06	2.05	1.91	2.17	2.29	2.09
mean (*ms*^−1^)	9.71	9.05	9.57	9.56	8.98	9.60
maximum (*ms*^−1^)	11.33	10.87	10.97	11.61	11.07	11.48

QuikSCAT parameters						

mean (*ms*^−1^)	8.92					
std. dev. (*ms*^−1^)	2.11					
maximum (*ms*^−1^)	11.2					

## References

[1] Adamo M., De Carolis G., Morelli S., Parmiggiani F. (2004). Synergic use of SAR imagery and high-resolution atmospheric model to estimate wind vector over the Mediterranean Sea. Proc. SPIE.

[2] Portabella M., Stoffelen A., Johannessen J.A. (2002). Toward an optimal inversion method for synthetic aperture radar wind retrieval. J. Geophys. Res.

[3] Cameron I., Lumsdon P., Walker N., Woodhouse I. Synthetic aperture radar for offshore wind resource assessment and wind farm development in the UK.

[4] Girard-Ardhuin F., Mercier G., Collard F., Garello R. (2005). Operational oil-slick characterization by SAR imagery and synergistic data. IEEE J. Oceanic Eng.

[5] Brekke C., Solberg A.H.S. (2005). Oil spill detection by satellite remote sensing. Remote Sens. Environ.

[6] Solberg A.H.S., Brekke C., Husøy P.O. (2007). Oil spill detection in RADARSAT and ENVISAT SAR images. IEEE Trans. Geosci. Remote Sens.

[7] Du Y., Vachon P.W., Wolfe J. (2002). Wind direction estimation from SAR images of the ocean using wavelet analysis. Canadian J. Remote Sens.

[8] Fichaux N., Ranchin T. (2002). Combined extraction of high spatial resolution wind speed and wind direction from SAR images: A new approach using wavelet transform. Canadian J. Remote Sens..

[9] Wackerman C.C., Rufenach C.L., Shuchman R.A., Johannessen J.A., Davidson K.L. (1996). Wind vector retrieval using ERS-1 synthetic aperture radar imagery. IEEE Trans. Geosci. Remote Sens.

[10] Zecchetto S., De Biasio F. (2002). On shape, orientation, and structure of atmospheric cells inside wind rolls in two SAR images. IEEE Trans. Geosci. Remote Sens.

[11] Ceccarelli M., De Filippo M., Di Bisceglie M., Galdi C. A texture based approach for ocean surface wind detection in SAR images.

[12] Koch W. (2004). Directional analysis of SAR images aiming at wind direction. IEEE Trans. Geosci. Remote Sens.

[13] Horstmann J., Koch W. (2005). Measurement of ocean surface winds using synthetic aperture radars. IEEE J. Oceanic Eng.

[14] Monaldo F.M., Thompson D.R., Beal R.C., Pichel W.G., Clemente-Colon P. (2001). Comparison of SAR-derived wind speed with model predictions and ocean buoy measurements. IEEE Trans. Geosci. Remote Sens.

[15] Zecchetto S., De Biasio F. (2008). A wavelet-based technique for sea wind extraction from SAR images. IEEE Trans. Geosci. Remote Sens.

[16] Horstmann J., Koch W., Lehner S., Tonboe R. (2000). Wind retrieval over the ocean using synthetic aperture radar with C-band HH polarization. IEEE Trans. Geosci. Remote Sens.

[17] Agency C.S. Satellite RADARSAT-1, Canadian Space Agency. http://www.asc-csa.gc.ca/eng/satellites/radarsat1.

[18] Isoguchi O., Shimada M. (2009). An L-band ocean geophysical model function from PALSAR. IEEE Trans. Geosci. Remote Sens.

[19] Choisnard J., Power D., Davidson F., Stone B., Howell C., Randell C. (2007). Comparison of C-band SAR algorithms to derive surface wind vectors and initial findings in their use marine search and rescue. Canadian J. Remote Sens.

[20] QuikSCAT data are produced by Remote Sensing Systems and sponsored by the NASA Ocean Vector Winds Science Team. www.remss.com/.

[21] Daubechies I., Combes J.M., Grossmann A., Tchamitchian P. (1989). Orthonormal bases of wavelets with finite support–connection with discrete filters. Wavelets.

[22] Koch W., Feser F. (2006). Relationship between SAR-derived wind vectors and wind at 10-m height represented by a meososcale model. Amer. Meteor. Soc.

[23] Stoffelen A., Anderson D. (1997). Scatterometer data interpretation: measurement space and inversion. J. Atmos. Oceanic Tech.

[24] Guiting S., Yijun H., Yijun H. (2006). Comparison of two wind algorithms of ENVISAT ASAR at high wind. Chin. J. Oceanol. Limnol.

[25] Hersbach H., Stoffelen A., de Haan S. The improved C-band geophysical model function CMOD5.

[26] Horstmann J., Koch W., Lehner S. (2004). Ocean wind fields retrieval from the advanced synthetic aperture radar aboard ENVISAT. IEEE Trans. Geosci. Remote Sens.

[27] Kim D., Moon W.M. (2002). Estimation of sea surface wind vector using RADARSAT data.

[28] Thompson D.R., Elfouhaily T.M., Chapron B. Polarization ratio for microwave backscattering from the ocean surface at low to moderate incidence angles.

[29] Mallat S.G. (1998). A Wavelet Tour of Signal Processing.

[30] Holschneider M., Kronland-Martinet R., Morlet J., Tchamitchian P., Combes J.M., Grossmann A., Tchamitchian P. (1989). A real-time algorithm for signal analysis with the help of the wavelet transform. Wavelets, Time-Frequency Methods and Phase Space.

[31] Shensa M. (1992). The discrete wavelet transform: wedding the à trous and Mallat algorithms. IEEE Trans. Signal Process.

[32] Dutilleux P., Combes J.M., Grossmann A., Tchamitchian P. (1989). An implementation of the “algorithme à trous” to compute the wavelet transform. Wavelets. Time-Frequency Methods and Phase Space.

[33] Bijaoui A., Starck J.L., Murtagh F. (1998). Image Processing and Data Analysis: The Multiscale Approach.

[34] Hong L., Guan Y., Zhang L. An à trous algorithm based threshold shrinkage denoising method for blood oxygen signal.

[35] Lee T.S. (1996). Image representation using 2D Gabor wavelets. IEEE Trans. Pattern Anal. Machine Intell.

[36] Cheng Y.S., Liang T.C. (1994). Rotational invariant pattern recognition using a composite circular harmonic and 2D isotropic Mexican-hat wavelet filter. Optics Commun.

[37] Kutter M., Bhattacharjee S.K., Ebrahimi T. Towards second generation watermarking schemes.

[38] Horstmann J., Koch W., Lehner S., Tonboe R. (2002). Ocean winds from RADARSAT-1 ScanSAR. Canadian J. Remote Sens.

[39] Oliveira J.G., Medeiros W.E., Tabosa W.F., Vital H. (2008). From barchan to domic shape: evolution of a coastal sand dune in Northeastern Brazil based on GPR survey. Brazilian J. Geophy.

